# Comparison of stroke volume changes of LiDCO™plus and Flotrac™ during postoperative hemodynamic optimization

**DOI:** 10.1186/cc9479

**Published:** 2011-03-11

**Authors:** MG Costa, T Cecconet, P Chiarandini, S Buttera, L Pompei, G Della Rocca

**Affiliations:** 1 University of Udine, Italy

## Introduction

Postoperative hemodynamic optimization (PHO) [1] can be performed with mini invasive devices that showed different level of agreement when compared with the pulmonary artery catheter [2]. The aim of the study was to evaluate the concordance on stroke volume index changes (ΔSVI) obtained from calibrated (LiDCO™plus) and uncalibrated pulse contour (Vigileo™) devices in a surgical patient cohort during early PHO.

## Methods

The setting was a prospective study in the ICU of a university hospital. Twenty-seven patients undergoing abdominal surgery and a PHO protocol were enrolled. We compared the paired SVI values obtained by the two devices 30 seconds before and 2 minutes after ending a volume challenge (VC) of HES 130/0.4 (3 ml/kg). In the protocol a SVI increase >5% after volume expansion defined a responder patient. Concordance of the response in terms of SVI direction of changes detected by each monitor (Vigileo-SVI and LiDCO-SVI) was analysed as proposed by Critchley and colleagues [3]. A Bland-Altman plot was used to define bias and accuracy between SVI obtained from the studied devices.

## Results

The mean bias between LiDCO-SVI and Vigileo-SVI was 1.16 ml/m^2 ^with SD of 12.51 ml/m^2^. The 95% limit of agreement was from -23.36 to 25.68 ml/m^2^. During all of the study period 47 VCs were administered. In eight out of 27 patients also 13 dobutamine tests were performed. The two devices showed the same direction of changes in 78% of the cases. In detail, they showed the same direction in 83% of cases after VC and in 62% of cases after dobutamine administration. Among the concordant data pairs, the devices agreed in 81% of cases to define responder and nonresponder and in 82% and 75% of cases after VC and dobutamine tests, respectively.

## Conclusions

LiDCO™plus and Vigileo™ tests during a PHO protocol identified the same direction of changes in 78% of cases. Among this 78%, the devices agreed both in 81% of cases to define responder and nonresponder.

## References

[B1] Pearse R Crit Care 2005 9 687 693

[B2] Hadiani M Crit Care 2010 14 R212 10.1186/cc9335 PMC3220011 21092290

[B3] Critchley LA Anesth Analg 2010 111 1180 1192 10.1213/ANE.0b013e3181f08a5b 20736431

